# Suboptimal geographic accessibility to comprehensive HIV care in the US: regional and urban–rural differences

**DOI:** 10.1002/jia2.25286

**Published:** 2019-05-20

**Authors:** Steven P Masiano, Erika G Martin, Rose S Bono, Bassam Dahman, Lindsay M Sabik, Faye Z Belgrave, Adaora A Adimora, April D Kimmel

**Affiliations:** ^1^ Department of Health Behavior and Policy Virginia Commonwealth University School of Medicine Richmond VA USA; ^2^ Department of Public Administration and Policy University at Albany‐State University of New York Albany NY USA; ^3^ Department of Health Policy and Management University of Pittsburgh Pittsburgh PA USA; ^4^ Department of Psychology Virginia Commonwealth University Richmond VA USA; ^5^ Departments of Medicine and Epidemiology University of North Carolina at Chapel Hill Chapel Hill NC USA

**Keywords:** HIV/AIDS, geography, travel time, access to care, rural, disparities

## Abstract

Achieving US state and municipal benchmarks to end the HIV epidemic and promote health equity requires access to comprehensive HIV care. However, this care may not be geographically accessible for all people living with HIV (PLHIV). We estimated county‐level drive time and suboptimal geographic accessibility to HIV care across the contiguous US, assessing regional and urban–rural differences. We integrated publicly available data from four federal databases to identify and geocode sites providing comprehensive HIV care in 2015, defined as the co‐located provision of core HIV medical care and support services. Leveraging street network, US Census and HIV surveillance data (2014), we used geographic analysis to estimate the fastest one‐way drive time between the population‐weighted county centroid and the nearest site providing HIV care for counties reporting at least five diagnosed HIV cases. We summarized HIV care sites, county‐level drive time, population‐weighted drive time and suboptimal geographic accessibility to HIV care, by US region and county rurality (2013). Geographic accessibility to HIV care was suboptimal if drive time was >30 min, a common threshold for primary care accessibility in the general US population. Tests of statistical significance were not performed, since the analysis is population‐based. We identified 671 HIV care sites across the US, with 95% in urban counties. Nationwide, the median county‐level drive time to HIV care is 69 min (interquartile range (IQR) 66 min). The median county‐level drive time to HIV care for rural counties (90 min, IQR 61) is over twice that of urban counties (40 min, IQR 48), with the greatest urban–rural differences in the West. Nationally, population‐weighted drive time, an approximation of individual‐level drive time, is over five times longer in rural counties than in urban counties. Geographic access to HIV care is suboptimal for over 170,000 people diagnosed with HIV (19%), with over half of these individuals from the South and disproportionately the rural South. Nationally, approximately 80,000 (9%) drive over an hour to receive HIV care. Suboptimal geographic accessibility to HIV care is an important structural barrier in the US, particularly for rural residents living with HIV in the South and West. Targeted policies and interventions to address this challenge should become a priority.

## 1 Introduction

Many United States (US) municipalities and states are implementing strategic plans to end HIV as an epidemic 1. In addition to meeting benchmarks such as percent virally suppressed and number of new cases, these plans typically promote improved health equity for people living with or at risk for HIV. Achieving these goals requires access to and use of comprehensive, coordinated HIV care (henceforth, HIV care) for all populations. Receipt of HIV care is associated with higher retention in care and improved viral suppression 2, which are critical for improved quality and length of life and for preventing HIV transmission 3. However, these outcomes may be limited by inadequate geographic access to HIV care: mounting evidence suggests that people living with HIV (PLHIV) who travel longer to receive care are less likely to be linked to care and achieve viral suppression 4, 5.

While national efforts seek to improve access to HIV care for all PLHIV 6, nationwide estimates for geographic access to HIV care have not been reported. Furthermore, the extent to which geographic access to HIV care may be lower for PLHIV in rural versus urban areas is unknown. This urban–rural disparity is an area of particular relevance given a growing rural and suburban population of PLHIV 7, 8, 9 but historical allocation of federal HIV prevention funds and Ryan White HIV/AIDS programme resources to urban centres 10, 11. Evidence is also limited on whether urban–rural patterns of geographic access to HIV care persist across regions, given variation in disease burden; health care infrastructure; and state policies, resources and geography. In this context, we estimated county‐level drive time and suboptimal geographic accessibility to HIV care across the contiguous US, focusing on regional and urban–rural differences.

## 2 Methods

We integrated publicly available data to estimate county‐level drive time to HIV care for adolescents and adults living with HIV in the 48 contiguous US states. The analytic approach involved three main analytic steps: (1) identifying HIV care service locations, (2) estimating one‐way drive time between population‐weighted county centroids and the nearest HIV care site for counties with at least five diagnosed HIV cases and (3) examining suboptimal geographic accessibility and urban–rural differences for each Census‐designated US region (Northeast, Midwest, South, West).

### 2.1 Data

Data used in this study came primarily from publicly available sources. For identifying HIV care sites, we used service addresses and grant numbers for recipients of US federal HIV care programme funding in 2015, available via: Health Resources and Services Administration (HRSA) Find Grants (https://datawarehouse.hrsa.gov/tools/findgrants.aspx), HRSA Data Portal (https://datawarehouse.hrsa.gov/tools/dataportal.aspx) and the HIV Testing Sites & Care Services Locator (https://www.hiv.gov/locator). We also included grantees of the Ryan White Part C Early Intervention Services programme (available via: Tracking Accountability in Government Grants (https://taggs.hhs.gov)), a national model for provision of comprehensive, coordinated HIV care. We leveraged information from all four databases since previous work suggests that use of a single database does not fully identify all HIV care sites 12; therefore, collectively, service locations of these grant recipients represent a full geographic snapshot of comprehensive, coordinated HIV care service provision nationally. To estimate drive time to HIV care, we additionally used population‐weighted county centroids (https://www.census.gov, 2010), and street network data (StreetMap Premium for ArcGIS, 2017 release). County‐level geographic boundary files 13 used to estimate and map drive time came embedded in the geographic analysis software. Finally, to estimate diagnosed HIV cases with suboptimal geographic access to HIV care and urban–rural differences, we used county‐level reports of adults and adolescents diagnosed and living with HIV by year‐end 2014 (hereafter, *diagnosed HIV cases*; https://aidsvu.org, 2014) and county‐level urbanicity (National Center for Health Statistics, 2013).

### 2.2 Analytic approach

#### 2.2.1 Identifying HIV care sites

Using both service addresses and grant numbers, we merged data from the four federal databases to identify sites that provided HIV care in 2015. HIV care was defined as the co‐located provision of both HIV core medical care (e.g. availability of providers who prescribe antiretrovirals) and support services (e.g. transportation assistance). After excluding administrative addresses (e.g. post office boxes) and removing duplicate service addresses, we confirmed provision of HIV core and support services through a manual online search of each potential HIV care site. All confirmed HIV care sites were geocoded to identify point locations using the Texas A&M University GeoServices geocoder platform (http://geoservices.tamu.edu/) 14. We also mapped the geocoded HIV care site locations – including 30‐mile Euclidian buffers – and diagnosed HIV cases in order to understand the geographic area covered by a given HIV care site, the potential for site choice among people diagnosed with HIV in a given county, and geographic areas that are potentially underserved.

#### 2.2.2 Estimating county‐level drive time to HIV care

We used ArcMap 10.5.1 (Environmental Systems Research Institute, Redlands, CA, USA) with Network Analyst to estimate drive time over the fastest one‐way route from each population‐weighted county centroid to the nearest HIV care site. County‐level drive time to HIV care was calculated for each of 2433 counties with at least five diagnosed HIV adult or adolescent cases (https://aidsvu.org, 2014). These 2433 counties report 922,508 individuals diagnosed with HIV (Northeast: 25%; South: 44%; Midwest: 12%; West: 19%), approximately 872,000 of whom (95%) live in urban counties nationally (Northeast: 98%; South: 92%; Midwest: 93%; West: 98%). National and regional county‐level drive times were summarized using medians and interquartile ranges (IQRs).

#### 2.2.3 Assessing suboptimal geographic accessibility, population‐weighted drive time and urban–rural differences

We defined suboptimal geographic accessibility as county‐level drive time to HIV care >30 min. This reflects a common threshold for assessing access to primary care in the US 15, and is one of HRSA's criteria for determining health professional shortage areas 16. We also examined a >60‐min threshold, which has been used as an accessibility threshold for rural areas or specialist providers 15. We calculated the frequency and percentage of US counties with suboptimal geographic accessibility to HIV care, nationally and by region. We also estimated the number of diagnosed HIV cases with suboptimal geographic accessibility to care, nationwide as well as by region and rurality, by summing county‐level diagnosed HIV cases for counties identified as having suboptimal geographic accessibility to HIV care 17. We examined urban–rural differences for HIV care site locations, county‐level drive time, and suboptimal geographic accessibility, nationally and by region. Urban counties were defined as those in metropolitan statistical areas with urban clusters of at least 50,000 population; rural counties comprised all non‐metropolitan counties (adapted from National Center for Health Statistics, 2013) 18. Lastly, we calculated county‐level drive times weighted by the number of diagnosed HIV cases in each county. These population‐weighted average drive times provide an approximation of the average individual‐level drive time when aggregated across states, regions or rurality; while county‐level drive time captures geographic accessibility and remains unchanged for a given county, population‐weighted drive times account for differing numbers of HIV cases across counties and can be used to make further comparisons across geographic units of analysis. Variation in population‐weighted drive time was quantified using population‐weighted standard deviations.

Data were summarized in Stata 14.2 and mapped using ArcMap 10.5.1. Tests of statistical significance were not performed because the analysis is population‐based.

## 3 Results

### 3.1 HIV care sites

**Figure 1:**
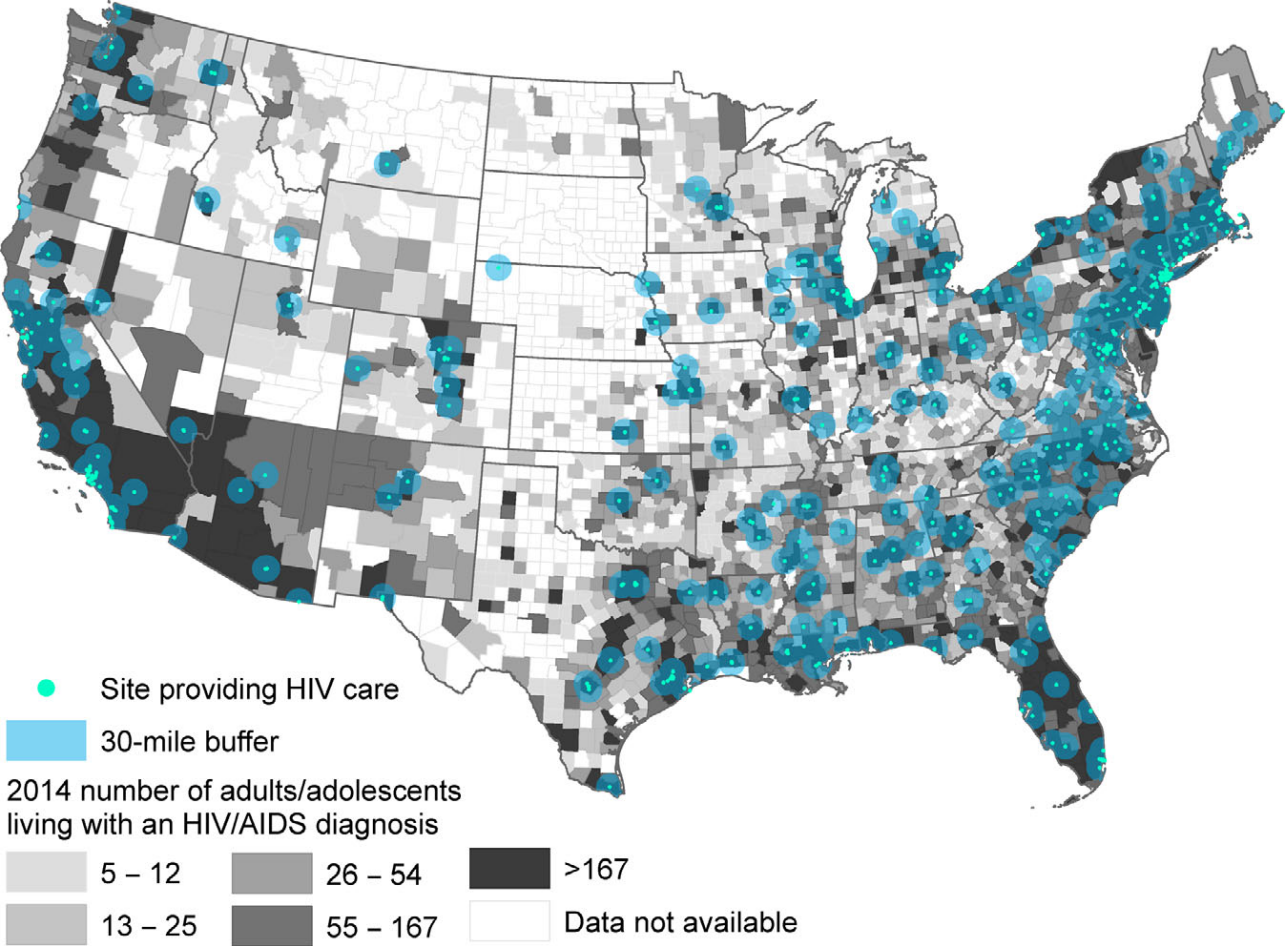
HIV care sites and diagnosed HIV cases in the contiguous United States This figure shows the locations of sites providing comprehensive, coordinated HIV care in the contiguous United States, for diagnosed HIV case quintiles. Surrounding the sites are catchment areas, defined as 30‐mile Euclidian buffers, which are shown in blue. For HIV case quintiles, darker shades of grey in the map reflect higher numbers of county‐level diagnosed HIV cases. While several areas along the East and West coasts have multiple HIV care sites that are geographically concentrated, many counties in the West and Midwest are more than 30 miles from the nearest HIV care site.

We identified 671 HIV care sites (Northeast: 30%; South: 34%; Midwest: 18%; and West: 18%). Nationally, 95% of HIV care sites are in urban counties, ranging from 91% in the South to 97% in the West, Midwest and Northeast regions. HIV care sites are geographically concentrated in areas with relatively high numbers of diagnosed HIV cases in the Northeast, as well as in select states in the West (e.g. California) and South (e.g. North Carolina), with all US regions having counties beyond the 30‐mile catchments of identified HIV care sites (Figure 1).

### 3.2 County‐level drive time to HIV care

Among counties with at least five diagnosed HIV cases, the median county‐level drive time between each population‐weighted centroid and the nearest HIV care site is 69 min (IQR 66 min) (Table 1). County‐level drive time varies by region, with the shortest county‐level drive time in the Northeast (median 40 min, IQR 48) and the highest in the Midwest (median 81 min, IQR 67). Nationally, median county‐level drive time for rural counties (90 min, IQR 61) is over twice that of urban counties (40 min, IQR 48), with the largest urban–rural difference in the West where median county‐level drive time for rural counties (136 min, IQR 113) is over triple that for urban counties (42 min, IQR 60). Within nearly every state, the median travel time was higher in rural counties compared with urban counties, although there was substantial variation between states in overall median drive time and in the size of urban–rural differences.

**Table 1:** Drive time to HIV care, by rurality^a^

	County‐level drive time to HIV care in minutes (median (interquartile range))^b^			Number of diagnosed HIV cases			Population‐weighted drive time (weighted standard deviation) in minutes^c^		
	All counties	Urban counties	Rural counties	All counties	Urban counties	Rural counties	All counties	Urban counties	Rural counties
All counties	69 (66)	40 (48)	90 (61)	922,508	872,156	50,352	20 (129)	16 (80)	82 (169)
West	80 (118)	42 (60)	136 (113)	177,278	173,220	4058	23 (162)	20 (104)	130 (181)
Arizona	101 (119)	57 (84)	155 (104)	14,233	13,878	355	93 (395)	92 (290)	147 (165)
California	41 (53)	22 (42)	74 (72)	119,077	118,353	724	13 (47)	12 (38)	81 (123)
Colorado	82 (133)	19 (37)	151 (126)	11,561	11,007	554	15 (61)	10 (26)	126 (202)
Idaho	78 (80)	55 (52)	117 (61)	809	647	162	35 (76)	19 (33)	101 (96)
Montana	245 (99)	129 (205)	248 (34)	467	259	208	166 (261)	109 (168)	237 (235)
Nevada	75 (136)	3 (45)	112 (140)	7698	7462	236	7 (19)	3 (4)	122 (113)
New Mexico	121 (138)	40 (52)	136 (146)	2845	2217	628	55 (129)	29 (45)	149 (176)
Oregon	102 (97)	69 (44)	140 (83)	5915	5490	425	42 (119)	34 (68)	145 (149)
Utah	100 (119)	66 (52)	166 (34)	2636	2545	91	33 (117)	30 (76)	133 (118)
Washington	78 (93)	48 (67)	109 (67)	11,802	11,251	551	24 (100)	20 (68)	109 (102)
Wyoming	182 (79)	163 (129)	182 (64)	235	111	124	173 (234)	133 (88)	209 (223)
Midwest	81 (67)	48 (54)	100 (63)	110,190	102,882	7308	24 (169)	19 (91)	97 (123)
Illinois	66 (58)	43 (43)	81 (45)	33,585	32,506	1079	10 (74)	8 (40)	79 (91)
Indiana	75 (48)	58 (42)	93 (37)	9681	8876	805	37 (164)	33 (111)	89 (94)
Iowa	87 (77)	38 (68)	103 (62)	2090	1609	481	48 (134)	34 (72)	94 (86)
Kansas	91 (80)	38 (38)	116 (61)	2883	2466	417	34 (83)	18 (33)	127 (131)
Michigan	73 (59)	47 (56)	80 (69)	15,241	14,465	776	30 (140)	26 (76)	102 (156)
Minnesota	98 (96)	58 (88)	126 (79)	7645	7177	468	28 (136)	22 (76)	126 (130)
Missouri	97 (66)	56 (56)	111 (58)	12,224	11,139	1085	26 (112)	18 (53)	110 (131)
Nebraska	112 (132)	18 (46)	156 (115)	1473	1402	71	22 (63)	15 (31)	162 (108)
North Dakota	343 (120)	297 (145)	356 (104)	264	190	74	272 (470)	229 (267)	383 (504)
Ohio	60 (47)	37 (26)	82 (38)	19,373	17,900	1473	22 (79)	18 (46)	70 (78)
South Dakota	–	–	–	–	–	–	–	–	–
Wisconsin	116 (94)	60 (80)	129 (87)	5731	5152	579	33 (145)	25 (81)	101 (114)
Northeast	40 (48)	23 (31)	65 (33)	232,537	226,930	5607	9 (34)	8 (23)	65 (119)
Connecticut	18 (11)	19 (13)	15 (–)	10,130	9948	182	16 (26)	16 (23)	15 (–)
Maine	51 (30)	37 (1)	64 (26)	1280	915	365	36 (54)	25 (29)	62 (61)
Massachusetts	21 (35)	18 (16)	61 (26)	18,724	18,578	146	12 (20)	11 (16)	51 (45)
New Hampshire	60 (35)	41 (32)	68 (36)	1203	782	421	49 (75)	43 (40)	60 (66)
New Jersey	10 (15)	10 (15)	–	37,435	37,435	–	8 (12)	8 (12)	–
New York	47 (46)	29 (41)	65 (55)	128,956	125,884	3072	7 (24)	6 (15)	70 (98)
Pennsylvania	48 (47)	36 (41)	70 (31)	32,129	31,111	1018	10 (46)	8 (29)	68 (86)
Rhode Island	21 (22)	21 (22)	–	2097	2097	–	11 (23)	11 (23)	–
Vermont	60 (35)	37 (37)	66 (29)	583	180	403	47 (48)	17 (22)	61 (46)
South	66 (59)	41 (43)	84 (50)	402,503	369,124	33,379	23 (128)	19 (77)	75 (155)
Alabama	64 (50)	54 (32)	73 (39)	12,097	10,384	1713	28 (81)	19 (43)	78 (98)
Arkansas	101 (79)	59 (103)	107 (62)	5138	3872	1266	53 (182)	42 (104)	88 (122)
DC	2 (–)	2 (–)	–	15,173	15,173	–	2 (–)	2 (–)	–
Delaware	71 (90)	71 (90)	–	3213	3213	–	37 (39)	37 (39)	–
Florida	54 (53)	37 (40)	80 (18)	102,756	99,219	3537	23 (76)	21 (58)	85 (144)
Georgia	66 (49)	47 (40)	80 (38)	39,597	35,444	4153	28 (134)	22 (85)	76 (90)
Kentucky	77 (60)	44 (45)	92 (55)	5931	4859	1072	29 (135)	17 (55)	87 (116)
Louisiana	57 (42)	38 (42)	69 (25)	19,829	17,920	1909	21 (64)	17 (41)	66 (88)
Maryland	46 (57)	38 (51)	86 (16)	31,540	31,238	302	12 (28)	11 (23)	96 (109)
Mississippi	56 (44)	44 (34)	62 (45)	8910	5080	3830	35 (93)	19 (41)	55 (77)
North Carolina	43 (43)	30 (31)	62 (31)	26,141	21,627	4514	21 (58)	15 (37)	48 (63)
Oklahoma	90 (67)	45 (32)	102 (62)	5411	4475	936	32 (118)	18 (42)	102 (112)
South Carolina	35 (28)	33 (39)	42 (35)	12,966	11,182	1784	22 (49)	20 (37)	38 (51)
Tennessee	83 (48)	65 (57)	100 (38)	15,813	14,492	1321	27 (132)	21 (78)	93 (92)
Texas	100 (103)	53 (94)	116 (92)	75,445	70,519	4926	26 (141)	20 (73)	109 (272)
Virginia	40 (43)	30 (33)	64 (27)	20,782	19,163	1619	17 (49)	14 (34)	59 (75)
West Virginia	76 (57)	59 (40)	102 (48)	1761	1264	497	50 (87)	35 (53)	89 (95)

HIV care, comprehensive, coordinated HIV care.

^a^Reported for counties in which there are at least five diagnosed HIV cases (https://aidsvu.org, 2014, accessed January 2018). State‐level diagnosed HIV cases were used for the District of Columbia. HIV surveillance data are not reported for South Dakota. New Jersey, Rhode Island, Delaware and DC have no counties classified as rural. For DC and Connecticut, (–) indicates that no interquartile range was calculated, as DC represents a single geographic entity and Connecticut has a single rural county. The urban–rural dichotomy was created from the 2013 six‐class taxonomy of urban status by the National Center for Health Statistics. Counties in metropolitan statistical areas with urban clusters ≥ 50,000 population were classified as urban while those in nonmetropolitan statistical areas (i.e. micropolitan or noncore counties) were classified as rural; ^b^county‐level drive time refers to one‐way drive time from the population‐weighted centroid of each county to the nearest HIV care site; ^c^population‐weighted drive time refers to county‐level drive times weighted by the number of diagnosed HIV cases in a given county. These population‐weighted drive times provide an approximation of the average individual‐level drive time when aggregated across states, regions or rurality.

### 3.3 Diagnosed HIV cases with suboptimal geographic accessibility to care

**Figure 2:**
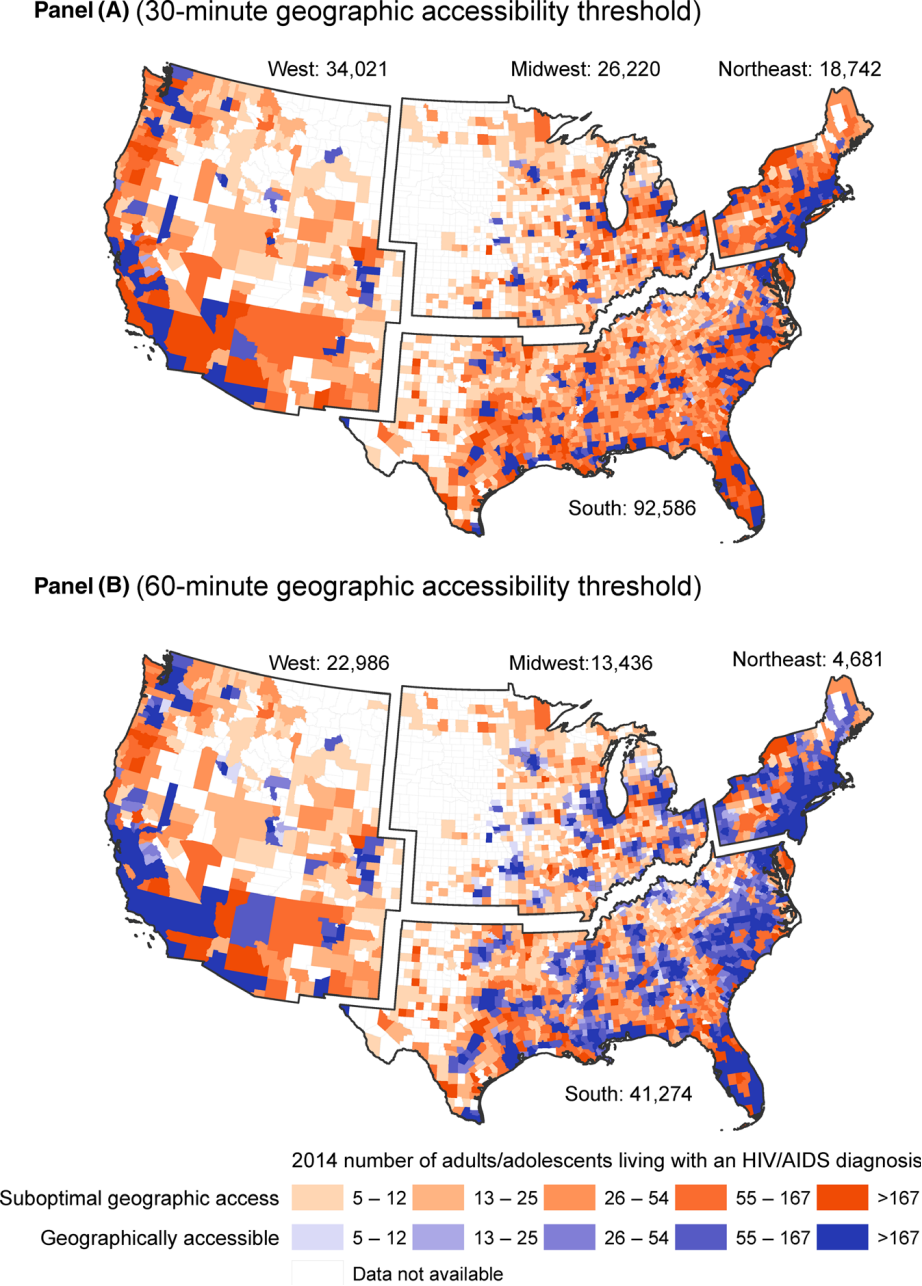
Diagnosed cases of HIV with suboptimal geographic access to HIV care, by US region These maps show counties with accessible (shown in blue) or suboptimal (shown in orange) geographic access to HIV care, by diagnosed HIV case quintile, for 30‐min (Panel A) and 60‐min (Panel B) geographic accessibility thresholds. Darker shades of each colour reflect higher numbers of county‐level diagnosed HIV cases. Numbers shown next to each region label reflect the number of diagnosed HIV cases, by region, living in counties for which geographic access to HIV care is suboptimal (i.e. requiring > 30 min (Panel A) or > 60 min (Panel B) one‐way drive time). Every US region contains counties with suboptimal geographic access to HIV care. Relative to other regions, the South is characterized by a large and widespread number of counties that have a higher HIV case burden with suboptimal geographic accessibility to HIV care. For a 30‐min geographic accessibility threshold, an estimated 171,569 (or nearly 1 in 5) Americans living with an HIV diagnosis have suboptimal geographic access to HIV care. Of these, over half (54%) are in the South, followed by the West (20%), Midwest (15%) and Northeast (11%). For a 60‐min geographic accessibility threshold, an estimated 82,377 Americans living with an HIV diagnosis have suboptimal geographic access to HIV care. Of these, approximately half are in the South, followed by the West (28%), Midwest (16%) and Northeast (6%). HIV care, comprehensive, coordinated HIV care; US, United States.

Geographic accessibility to HIV care is suboptimal for 1995 (82%) of US counties and 171,569 (19%) of people living with diagnosed HIV (Figure 2). Most of the diagnosed individuals with suboptimal geographic accessibility to HIV care live in the South (54%), followed by the West (20%), Midwest (15%) and Northeast (11%). Twenty‐seven percent of diagnosed individuals with suboptimal geographic accessibility to HIV care live in rural US counties, although 5% of people with an HIV diagnosis live in rural counties nationally. The South has the highest percentage of diagnosed cases with suboptimal geographic accessibility in rural counties of any region (32%), with nearly two‐thirds of individuals with suboptimal geographic access to HIV care from rural Southern counties (Table 2).

**Table 2:** Distribution of diagnosed HIV cases with suboptimal geographic accessibility to HIV care^a^

	Urban^b^		Rural^b^	
	Frequency	Percent	Frequency	Percent
Total	125,877	73	45,692	27
Northeast	13,856	74	4886	26
South	62,715	68	29,871	32
Midwest	19,247	73	6973	27
West	30,059	88	3962	12

^a^Suboptimal geographic accessibility is defined as drive time > 30 min from the population‐weighted county centroid to the nearest site of comprehensive, coordinated HIV care; ^b^counties in metropolitan statistical areas with urban clusters > 50,000 population are classified as urban, while those in nonmetropolitan statistical areas (i.e. micropolitan or noncore counties) are classified as rural.

When using a higher threshold for suboptimal geographic accessibility (60 min vs. 30 min drive time), the number of diagnosed HIV cases with suboptimal geographic access to HIV care decreases to 82,377 (or nearly 1 in 10 living with diagnosed HIV). Regional differences in suboptimal geographic accessibility to HIV care decrease: 50% of individuals with suboptimal geographic accessibility to HIV care live in the South, followed by the West (28%), Midwest (16%) and Northeast (6%). Urban–rural differences also decrease, with 39% of individuals with suboptimal geographic accessibility to HIV care living in rural counties, most of whom live in the South (62%).

### 3.4 Population‐weighted drive time to HIV care

The population‐weighted drive time, or the county‐level drive time weighted by the number of diagnosed HIV cases in each county, is over five times longer in rural counties (82 min) than in urban counties (16 min) (Table 1). Urban–rural differences in population‐weighted drive time vary by region, ranging from a fourfold difference in the South to an over eightfold difference in the Northeast. Similarly, state‐level differences are wide, with urban–rural differences in population‐weighted drive time to care as small as 0.9 times in Connecticut (Northeast region) to nearly 41 times in Nevada (West).

## 4 Discussion

This is the first effort to quantify geographic accessibility to HIV care across the contiguous US and examine differences by region for rural versus urban residence. Nationally, nearly 1 in 5 Americans diagnosed with HIV reside in a county with suboptimal geographic accessibility to HIV care, travelling more than 30 min to access care. All US regions have residents for whom geographic access to HIV care is suboptimal. Over half of individuals with suboptimal geographic accessibility to HIV care live in the South – and, disproportionately, the rural South – yet the greatest urban–rural differences in drive time are in the West.

Geographic access to care differs markedly across US regions, and each region may require unique solutions. For example, in the South, a region with a growing HIV burden, particularly in rural areas 19, both urban and rural residents living with HIV identify transportation as a significant barrier to care, contributing to missed medical visits and antiretroviral therapy doses 20. This region has the largest share of diagnosed HIV cases for which geographic access to care is suboptimal, so alternative solutions (e.g. mobile HIV clinics) that reduce transportation challenges could be useful. Residents of other regions confront suboptimal geographic accessibility to HIV care as well. The highest county‐level drive time to care is in the Midwest; urban–rural differences in county‐level drive time are most striking in the West, where the median county‐level drive time for rural residents is over two hours, compared to approximately 40 min for those who live in urban counties. Here, telemedicine and telehealth collaborations between specialists and primary care providers may help address suboptimal geographic accessibility to HIV care. These models have shown promise for rural veterans with HIV 21 and expanded access to specialists for individuals with complex, chronic diseases 22. HIV care through federally qualified health centres – publicly‐funded community clinics offering primary and preventive health care to low‐income populations – also has the potential to address geographic barriers to care 12. While more research is needed prior to widespread implementation of any solution, the consistent finding of limited geographic accessibility in rural areas across all US regions highlights the need to reconsider patterns of HIV prevention and treatment funding that historically have allocated funds almost exclusively to urban areas.

Findings from this analysis build on an emerging literature examining geographic access to care for PLHIV in the US. Evidence from two major urban centres in the US suggests that PLHIV travel an average of 4.4 miles to their HIV care provider in Philadelphia 23, while median commute time to HIV care is 22 min among men who have sex with men in Atlanta 24. County‐level estimates of drive time to HIV care from urban counties, in the current analysis as well as in previous work focused on the US South 12, are much greater. This difference may be due to the fact that we estimated drive time at the county level rather than at the individual level. However, these differences are more likely because the current work captures drive times to HIV care for many urban communities, including those that are in less densely populated metropolitan areas, instead of for single super‐urban cities that may not generalize to all urban locales in the US. This work corroborates notable urban–rural disparities in geographic accessibility to HIV care: rural veterans living with HIV may need to travel much longer to reach primary or specialty HIV care compared to urban veterans living with HIV 25. Such urban–rural differences in geographic accessibility to HIV care may influence care engagement among PLHIV in urban versus rural US communities 26. Furthermore, while evidence is not available on the relationship between geographic accessibility to care and HIV outcomes to our knowledge, longer travel times or distances to care have been associated with worse health outcomes for other complex, chronic diseases 27, 28, suggesting that suboptimal geographic accessibility could contribute to urban–rural disparities in HIV health outcomes.

These findings describe geographic accessibility of HIV care at a single point in time within the rapidly evolving US healthcare and policy environment. US federal discretionary spending for Ryan White programmes, a key source of comprehensive, coordinated HIV care, has remained relatively flat in recent years 10, 29, while the total number of HIV cases has increased 9. These trends have likely reduced per capita spending for HIV, limited HIV care service availability, and decreased the number of facilities providing comprehensive, coordinated HIV care. Meanwhile, ongoing debate over the Affordable Care Act leaves the availability of comprehensive insurance coverage and Medicaid expansion uncertain, potentially limiting the ability of PLHIV to access care in local networks. Yet improving geographic accessibility of care remains a current national priority. In January 2019, the Department of Veterans Affairs, which provides care to over 30,000 veterans living with HIV 30, proposed new standards for access to care which would allow veterans who drive more than 30 min to reach primary care or 60 min to reach specialty care to seek care at private facilities 31. Ultimately, while the drive times reported in this study may not precisely reflect the current context, they provide important evidence using a recent snapshot of HIV care availability.

This analysis has limitations. First, drive time to HIV care may be underestimated for individuals in some urban counties as we were unable to account for public transportation options due to inconsistent data availability, thereby potentially overestimating some urban–rural differences. We also did not have individual‐level data for the residences of people diagnosed with HIV, and therefore used population‐weighted county centroids in place of individuals’ residences. This use of a single origin point masks individual variation in drive time, although for different geographic units of analysis, variation in individual‐level drive time can be approximated by population‐weighted drive time estimates. Second, HIV care sites in our sample do not include sites providing comprehensive, coordinated HIV care that do not receive federal funding. However, the large percentage of PLHIV who have lower incomes and may rely on federally funded programmes to support care delivery 32 suggests that the sample used in the current analysis adequately captures sites providing comprehensive, coordinated HIV care in the US. Further, although our analysis does not include sites primarily serving veterans, our results complement existing literature suggesting that rural veterans living with HIV generally have longer drive times to primary and infectious disease specialty care delivered by the Department of Veterans Affairs 25. Third, while we selected a 30‐min threshold for comparability to existing primary care thresholds, we acknowledge that there is no universally accepted threshold for suboptimal geographic accessibility in the US, and that geographic accessibility thresholds appropriate for the US may not apply in other settings with different resources, health care systems and geographies. Fourth, our estimates of drive time assume that individuals visit the closest HIV care site and can use a personal vehicle, and they do not account for other care availability characteristics or overlapping service catchment areas. Future research should expand upon our initial inquiry as more detailed and widely available provider‐level data become available, and should use alternative spatial accessibility approaches that capture provider availability and accessibility, as well as demand for HIV services, in a single metric. Fifth, we were unable to estimate drive time using a smaller unit of geographic analysis (e.g. ZIP code tabulation area) due to limited and inconsistent data availability on diagnosed HIV cases at a smaller geographic unit. Finally, the number with suboptimal geographic access to HIV care, particularly in rural counties, may be underestimated since cases are reported only for counties with at least five diagnosed HIV cases.

## 5 Conclusions

In summary, we find that suboptimal geographic access to HIV care is a critical structural barrier in the US, particularly for those living in rural communities in the South and West. While the path to eliminating HIV in the US is within reach 33, doing so will require continuous access to and utilization of comprehensive HIV care, which is vital for achieving and maintaining viral suppression. With nearly half of PLHIV not virally suppressed 34, making progress towards national goals that will end the epidemic and improve health equity remains a national priority. Our findings demonstrate room for improvement in one measure of access to HIV care, geographic accessibility.

## Competing interests

The authors declare no competing interests.

## Authors’ contributions

ADK conceived and designed this study. SPM conducted the analysis. SPM, EGM, RSB, BD and ADK interpreted the findings. SPM, RSB and ADK drafted the initial manuscript and RSB and ADK revised subsequent versions of the manuscript. EGM, BD, LMS, FZB and AAA provided critical feedback on the manuscript. SPM, EGM, RSB, BD, LMS, FZB, AAA and ADK approved the final manuscript.
